# The passage of a diffusible indicator through a microvascular system

**DOI:** 10.1186/1742-4682-10-10

**Published:** 2013-02-13

**Authors:** Victor V Kislukhin

**Affiliations:** 1 Transonic System, Inc, 34 Dutch Mill Road, Ithaca, N.Y, 14850, USA

**Keywords:** Mathematical model, Microcirculation, Permeability, Poisson distribution

## Abstract

The aim. (1) To develop a mathematical model of the passage of a diffusible indicator through microcirculation based on a stochastic description of diffusion and flow; (2) To use Goresky transform of the dilution curves of the diffusible indicators for the estimation of the permeability of a tissue-capillary barrier.

The method. We assume that there are two causes for flow to be stochastic: (a) All microvessels are divided between open and closed microvessels. There exists random exchange between the two groups. (b) The flow through open microvessels is also random. We assume that each diffusing tracer has a probability to leave the intravascular space, and has a probability to return. We also assume that all considered processes are stationary (stability of microcirculation).

Conclusion. (a) The distribution of the time to pass microcirculation by diffusing indicator is given by a compound Poisson distribution; (b) The permeability of tissue-capillary barrier can be obtained from the means, delay, and dispersions of the dilutions of intravascular and diffusing traces.

## Introduction

From the beginning the theory of the passage of a tracer through an organ exploits two approaches. The first approach considers the vascular system as a black box characterized by a probability density function of a transit time (the transport function). It started with the work of Stephenson [1]. He suggested that a dilution curve is a distribution of the time it takes for an indicator to pass through an organ. His approach was further developed by Meier & Zierler [2]. They detailed the relationship among mean transit time, flow, and blood volume. Thus was established the foundation of the indicator dilution theory and its practical application. The problem with the black box approach was formulated by Zierler [3]: “What mechanism shapes the transport function?”

At the same time, there are attempts to reveal and/or impose on the vascular architecture and irregularities of flow such properties that lead to the transport function as one of the well-known probability density functions. The used distributions are from the random walk [4] to the mixing chambers [5,6]. Intensive review of the transport functions can be found in [7,8]. The main problem with a distribution chosen in advance is that the physiological background of transport function remains unclear.

In his quest to select from different math models such that generate a transport function for a liver’s circulation Goresky [9] found out that by the transformation of any dilution curves (from RBC-Cr^51^ to the DHO) by dividing time by the corrected mean transit time (MTT - delay), and simultaneously multiplying the ordinate of the dilution by the same factor, one get all dilution curves coincided. This discovery generated the hope for better understanding of microcirculation, as it was said by Zierler [3]: “It seemed to telling us something new about what is inside the black box”.

Thus the aim of the manuscript is

(a) To present a mathematical model of the passage of a diffusing tracer through microcirculation.

(b) To use Goresky transform for estimation of the permeability of a tissue-capillary barrier.

### Description of diffusion

Let r be the time spent by a diffusing particle within a microcirculation. Let denote as s the time that a particle spends being within the intravascular space, thus the time r-s is the time the particle spends in the extravascular space. Also, assume that two probabilities of exchange, from intra to extra vascular space and back, are constant. Accepted properties of the exchange mean that there exist two exponential distributions: (a) A distribution to leave a vascular space with density *δ* ·  exp(−*δt*), where 1/*δ* is the average time for the particle, being in a microcirculation, to flux to the extravascular space (thus 1/*δ* is the intensity to pass endothelial barrier); (b) A distribution to return into vasculature with density *f*_*γ*,1_(*t*) = *γ* ·  exp(−*γt*) where 1/*γ* is the average time for a particle to be in the extravascular space before its return into a vascular space. We denote as *f*_*γ*,0_(*t*) = 1 if time t=0 and *f*_*γ*,0_(*t*) = 0 if t>0. The exponential law for the time to be within a microcirculation (between two consecutive jumps) leads to the number of jumps out of a capillary during time s (before leaving microcirculation) to be Poisson distributed with probability to have n jumps as $p_{n}=\exp\left(-\delta \cdot s\right)\frac{{\left(\delta \cdot s\right)}^{n}}{n!}$. Since the n jumps out of capillary space are connected with n returns out of extravascular space with the distribution *f*_*γ*,*n*_(*t*) = (*γt*)^*n* − 1^*γ* ·  exp(−*γt*)/(*n* − 1) !, the time to be in extravascular space, under condition that the time to be in intravascular space is s, and follows to a compound Poisson distribution [10]:

(1) $$D\left(r,s\right)=\sum_{n=0}^{\infty }p_{n}\cdot f_{\gamma ,n}\left(r-s\right)=\{\begin{array}{c} 0\;r<s\\ e^{-\mathrm{\delta s}}\;r=s\\ \sum_{n=1}^{\infty }\frac{{\left(\delta \cdot s\right)}^{n}}{n!}e^{-\mathrm{\delta s}}\frac{\gamma^{n}}{\left(n-1\right)!}{\left(r-s\right)}^{n-1}e^{-\gamma \left(r-s\right)}r>s \end{array}\;$$

The Laplace transform of D(r,s)

(2) $$d\left(\lambda ,s\right)=\int \exp\left(-\mathrm{\lambda r}\right)\sum_{n=0}^{\infty }\frac{{\left(\delta \cdot s\right)}^{n}}{n!}\exp\left(-\mathrm{\delta s}\right)f_{\gamma ,n}\left(r-s\right)\mathrm{dr}=\exp\left(-\mathrm{\lambda s}\left(\frac{\gamma +\delta +\lambda }{\gamma +\lambda }\right)\right)$$

The equation analogous (1), as the solution of a diffusion equation (named as the Sangren-Shepard equation), is presented in Goresky et al. [11] in their attempt to explain Goresky phenomenon.

The time s in (1) and (2) is the time to pass through microcirculation, and thus it is variable. We assume that intravascular space both type of tracers (diffusing and intravascular) pass having the same distribution of the time s. Thus, our next step is the description of the passage of intravascular indicator.

### The passage of an intravascular indicator

The distribution of the s is a composition of two processes (a) the change of the state of any microvessel, meaning that closed microvessels become open and vice versa, this process will be denoted as vasomotion; and (b) a variation of the time to pass through microvessels being in the open state. Thus the time s is the sum of times s-T and T, where time s-T is the time spent by particles in the temporally closed microvessels and T is the time to pass through microvessels being in open state. T and s-T are mutually independent random variables. The T as variable depends on a tortuosity of microvessels and a heterogeneity of flow.

To find the distribution of the s we start with vasomotion. The time between interruptions of flow follows to the exponential distribution: *β* ·  exp(−*βt*) with 1/*β* as the average number of interruptions per unit of time, and the time needed for resuming of flow follows to the distribution *f*_*μ*,1_(*t*) = *μ* exp(−*μt*) where 1/*μ* is the mean time of being stopped. Thus the probability to have n stops is *p*_*n*_ = exp(−*βT*)(*βT*)^*n*^/*n* !. The conditional density of the time to pass through an organ by an intravascular indicator, V(s,T), with T arbitrary but fixed, is :

(3) $$V\left(s,T\right)=\exp\left(-\mathrm{\beta T}\right)\cdot \sum_{n=0,\infty }\frac{{\left(\mathrm{\beta T}\right)}^{n}}{n!}f_{\mu ,1}^{n*}\left(s-T\right)$$

Laplace transform of (3) is (4). The T in (4) is, actually, the variables and its distribution is denoted as G(T) with Laplace transform *g*(*λ*).

(4) $$v\left(\lambda ,T\right)=\int \exp\left(-\mathrm{\lambda S}\right)\sum_{n=0}^{\infty }\frac{{\left(\mathrm{\beta T}\right)}^{n}}{n!}e^{-\mathrm{\beta T}}f_{\mu ,1}^{n*}\left(S-T\right)\mathrm{dS}=\exp\left(-\mathrm{\lambda T}\left(\frac{\mu +\beta +\lambda }{\mu +\lambda }\right)\right)$$

The randomization of (4) by G(T) and the specificity of *v*(*λ*, *T*) lead to the Laplace transform of unconditional distribution to pass through microvasculature:

(5) $$v\left(\lambda \right)=\int \exp\left(-\mathrm{\lambda T}\left(\frac{\mu +\beta +\lambda }{\mu +\lambda }\right)\right)G\left(T\right)\mathrm{dT}=g\left(\lambda \frac{\mu +\beta +\lambda }{\mu +\lambda }\right)$$

### The passage of a diffusing indicator

We have a conditional distribution of s (the time T is arbitrary but fixed), thus the conditional Laplace transform of the distribution for a diffusible indicator:

(6) $$\begin{array}{ll} d(\lambda ,T) & =\int \exp\left(-\mathrm{\lambda s}\left(\frac{\gamma +\delta +\lambda }{\gamma +\lambda }\right)\right)\cdot V(s,T)ds=\exp\left(-\phi \left(\lambda \right)T\frac{\beta +\mu +\phi \left(\lambda \right)}{\mu +\phi \left(\lambda \right)}\right)=\\ & =\exp\left(-\mathrm{T\varphi}\left(\phi \left(\lambda \right)\right)\right) \end{array}$$

where $\phi \left(\lambda \right)=\lambda \cdot \frac{\gamma +\delta +\lambda }{\gamma +\lambda }$; and $\varphi \left(\lambda \right)=\lambda \left(\frac{\beta +\mu +\lambda }{\mu +\lambda }\right)$

To get the unconditional Laplace transform for the distribution of a diffusible indicator all we need is the randomization of (6) by the distribution of T [10]:

(7) $$d\left(\lambda \right)=\int \exp\left(-\mathrm{T\varphi}\left(\phi \left(\lambda \right)\right)\right)G\left(T\right)\mathrm{dT}=g\left(\varphi \left(\phi \left(\lambda \right)\right)\right)$$

### Goresky transform

The essence of Goresky phenomenon [9] can be expressed as follow: by transformation of any dilution curve (from RBC-Cr^51^ to the DHO) by dividing time by the mean transit time (MTT) corrected by common delay, T, and simultaneously multiplying the ordinate of a dilution by the same factor, one would get all dilution curves simultaneously coincided. Let denote this transform as Goresky transform.

Formally **Goresky transform** is performed in the two steps

(a) Obtaining of the coefficient **a** of the transform:

(8) $$a\left(M_{V}-T\right)=M_{D}-T;a=\frac{M_{D}-T}{M_{V}-T}$$

where M_V_ is the mean time to pass through the investigated tissue by the intravascular indicator, M_D_ is the mean time to pass through by diffusing indicator, and T is the common delay;

(9) $$D_{\mathrm{GT}}\left(t\right)=\mathrm{aD}\left(T+\left(t-T\right)/a\right),t>T;D_{\mathrm{GT}}\left(t\right)=0,t=T,\mathrm{or}\;t<T$$

Thus we obtain the Goresky coefficient, a, and the new shape, D_GT_(t), for the dilution curve of the diffusing indicator.

(b) The distribution of the diffusing indicator, D(t), changes to distribution D_GT_(t):

### Goresky phenomenon

If two distributions, F(t) and G(t) coincide then all their moments *M*_*k*_ = (−1)^*k*^*f*^(*k*)^(0), where f(λ) is the Laplace transform of F(t), are equal, M_k_(F)=M_k_(G), for each k. The practical coincidence can be reached by equalities only of the first two moments, or, what is the same, the equality of the means and dispersions. In our case we have two dilution curves, from the intravascular indicator, V(t) and, after Goresky transform, D_GT_(t), the dilution curve obtained from the diffusing indicator, D(t). Thus the Goresky phenomenon takes place if applying Goresky coefficient, see (8), we obtain next relation between dispersions of the intravascular (*D*_*V*_^2^) and diffusing indicators (*D*_*D*_^2^): *D*_*D*_^2^ = *a*^2^*D*_*V*_^2^; by other words, dispersions of V(t) and D_GT_(t) are equal.

### Permeability by Goresky transform

Our diffusing indicator has a distribution with Laplace transform *d*(*λ*) = *g*(*φ*(*ϕ*(*λ*))) (7), and a dilution of an intravascular indicator has Laplace transform *v*(*λ*) = *g*(*φ*(*λ*)) (5). Due to stochastic description of the diffusion one has $\phi \left(\lambda \right)=\lambda \cdot \frac{\gamma +\delta +\lambda }{\gamma +\lambda }$.

With such a presentation of *ϕ*(*λ*) Goresky transform leads to the determination of the characteristics of permeability of endothelial barrier, these are δ and γ. Also will be found out that the specificity of *g*(*λ*) and *φ*(*λ*) play no role (but *φ*(*λ*) should be infinitely divisible).

Indeed, the mean and dispersion of the diffusible indicator are

(10) $$M_{D}=M_{V}\left(1+\frac{\delta }{\gamma }\right);D_{D}^{2}=D_{V}^{2}{\left(1+\frac{\delta }{\gamma }\right)}^{2}+M_{V}\frac{2\delta }{\gamma^{2}};$$

Where M_v_ and D_v_ are mean and dispersion for the intravascular indicator. The (10) follows from next equations that established connection between M and D^2^ of any distribution, f(t), and derivatives of its Laplace transform *F*(*λ*) = ∫ exp(−*λt*)*f*(*t*)*dt*. Thus *M* = *F*^′^(*λ*)|_*λ* = 0_; and *D*^2^ = *F*″(*λ*)|_*λ* = 0_ − *M*^2^.

Now, if we put relations between two means, given by (10) into equation (8), we get, for Goresky coefficient, next equality:

(11) $$\;a=\frac{M_{D}-T}{M_{V}-T}=\frac{M_{V}\left(1+\frac{\delta }{\gamma }\right)-T}{M_{V}-T}$$

Since M_D_, M_V_, and T are known, the a can be calculated. Thus the knowledge of Goresky coefficient, a, leads to obtaining of *δ*/*γ*:

(12) $$\frac{\delta }{\gamma }=\left(a-1\right)\frac{\left(M_{V}-T\right)}{M_{V}};$$

The use of the second relation in (10), assuming the knowledge of dispersions, leads to the obtaining of γ:

(13) $$\gamma =\frac{2\delta \cdot M_{V}}{\gamma \cdot D_{V}^{2}}{\left(\left(\frac{D_{d}^{2}}{D_{V}^{2}}-{\left(1+\frac{\delta }{\gamma }\right)}^{2}\right)\right)}^{-1}$$

### Experiments with Goresky transform

**Figure 1:**
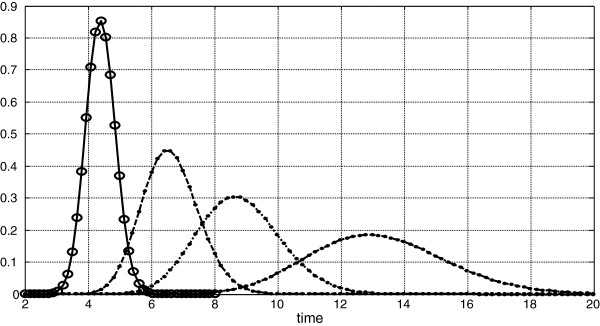
Four dilution curves, one is from non-diffusible indicator and three dilutions are from diffusible indicators, with different extravascular distribution, but with the same dispersions after Goresky transform.

On Figures 1 through 4 there are the experiments on PC with the math model of intravascular and diffusing indicators.

The distribution to pass through intravascular space is characterized by delay of 2 sec, and by binomial distribution given on N=40 points between 2 and 8 sec, thus with step h = 0.15 sec, and p=0.4 thus $p_{i}=\frac{40!}{i!\left(40-i\right)!}p^{i}{\left(1-p\right)}^{40-i}$. So the mean transit time M_v_=Nph+T=2.4+2=4.4; and dispersion D_v_^2^=Np(1-p)[h]^2^ = 0.216.

Distributions of diffusing indicators are additionally characterized by relation of extravascular/intravascular distribution, this is *δ*/*γ*. Thus we have relation between two means to pass through microcirculation (diffusing and intravascular): *M*_*d*_ = *M*_*v*_(1 + *δ*/*γ*). For the experiment are chosen three types of diffusing indicator, with *δ*/*γ* equal 0.5, 1.0 and 2.0 (by other words with small, medium and expanded extravascular space). Goresky transform leads to the next three, corresponding Goresky coefficients obtained by applying (8): they are 1.8; 2.7, and 4.4 correspondingly.

**Figure 2:**
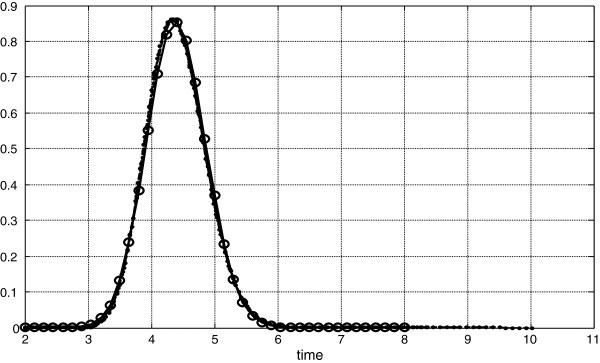
**Dilution curves from Figure**1** after Goresky transform.**

In common case dispersions are not equalized by Goresky coefficient so *a*^2^*D*_*V*_^2^ ≠ *D*_*D*_^2^. However, if we chose γ to fulfill equality between dispersion: *D*_*D*_^2^ = *a*^2^*D*_*V*_^2^; then Goresky transform leads to the dilutions given on Figure 2. On Figure 1 there are four initial dilution curves. Corresponding δ and γ are: for the second curve 7.2, 14.3, for the third curve 10.1, 10.1, and for the fourth curve 12.8, 6.4. Thus given characteristics of the permeability lead to the Goresky phenomenon.

**Figure 3:**
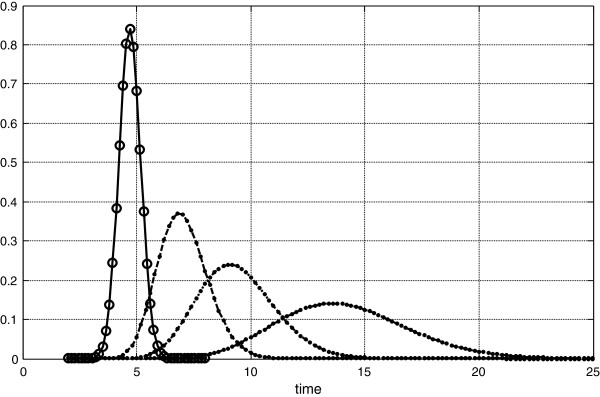
Four dilution curves, one is from non-diffusible indicator and three dilutions are from diffusible indicators, with different extravascular distribution, and with the dispersions not equal to dispersion of intravascular indicator after Goresky transform.

**Figure 4:**
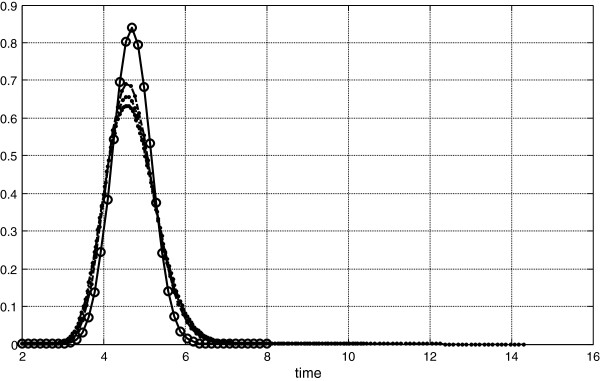
**Dilution curves from Figure**3** after Goresky transform.**

Figure 3 presents the same intravascular dilution but diffusing indicators are different. The probabilities to return into intravascular space are lesser than presented on Figure 1, by 2.5 times, thus dispersions of passing through microcirculation are increased and application of Goresky transform does not lead to the coincidence dilution curves, Figure 4. In this case corresponding δ and γ are: for the second curve 7.2, 5.7, for the third curve 10.1, 4.0, and for fourth curve 12.8, 2.6.

## Discussion

Equation (7) that gives Laplace transform of the distribution to pass through microcirculation by a diffusing indicator, and also (11) with (12) that give the expression for permeability obtained by Goresky transform are the main result of the manuscript. These equations are obtained by the exploiting exponential distributions of the times such as being in extra/intra vascular space, or being in temporally closed/open microvessels. The physical (physiological) assumptions leading to exponential distribution are based on the randomness of the passage of particles through microcirculation. The randomness is taken as stationary, indicating the constancy of parameters of a transport functions. Additionally, the trajectory of any particle follows markovian property, meaning that the future trajectory depends only on the current place of particle, and not on its past state. The base for such assumptions is (a) The force applied to the small volume containing a particle (the local pressure gradient) is not zero then the move exists, and velocity is V=k*F. This is plausible since in microcirculation Newton’s law: acceleration is equal sheer stress (viscosity force) minus gradient of pressure, can be simplified since the convective inertia can be ignored, Fung, [12]. (b) The random walk presentation of diffusion used to obtain (1) is a good approximation of diffusion expressed in partial deviations [13].

Existing methods of estimation of permeability of an endothelium are based on assumptions that vary from the negligible back diffusion to the very high permeability such that equilibrium, capillary-tissue is established instantly, see review [14]. Thus the Goresky transform for estimation of permeability could have some advantage since it is based only on assumptions of stochasticity of blood flow and diffusion.

## Conclusion

1. The markovian property of flow and diffusion lead to compound Poisson distribution for the time to pass through an organ, and thus to the composite functions for Laplace transform of diffusing and intravascular indicator.

2. The Goresky transform could be used for the estimation of permeability of the tissue/capillary barrier.

## Abbreviations

*r*: is the time to pass through microcirculation by a diffusing particle; *s*: is the time to pass through microcirculation by an intravascular particle; *r-s*: is the time spent by a diffusible particle in the extravascular space; T: is the time spent in open microvessels. Thus **s-T** is the time spent in closed vessels; t: is used as argument for any time-dependent process; denotation V(t . ): is used for the distribution of an intravascular indicator; denotation D(t . ): is used for the distribution of a diffusing indicator; T: is used as the denotation for a delay in section “Goresky transform” and section “Experiment with Goresky transform”.

## Competing interests

The author declares he has no competing interests.
